# Supplementary material for: Sickle cell disease complications: Prevalence and resource utilization
Source: PLoS One. 2019 Jul 5;14(7):e0214355. doi: 10.1371/journal.pone.0214355 (PMC6611562; doi:10.1371/journal.pone.0214355)
Supplement: S1 Table — SCD: Sickle cell disease. (DOCX) [file pone.0214355.s001.docx]

**S1 Table. List of SCD Complications.** SCD: Sickle cell disease.

| **Systems** | **Types of diseases** | **ICD-9-Codes** |
| --- | --- | --- |
| Cerebrovascular | Stroke | 430, 431, 433.x1, 434.x1, 436.x |
|  | Transient ischemic attack | 435.x |
|  | Seizures | 345.0x-345.5x, 345.7x-345.9x, 780.39 |
| Hepatic | Bilirubinemia | 782.4 |
|  | Gallstones | 574.x |
|  | Cholecystitis | 574.00, 574.01, 574.30, 574.31, 574.60, 574.61, 575.0, 575.1x |
|  | Biliary sludge | 576.8 |
|  | Acute choledocholithiasis | 574.5x |
|  | Acute hepatic sequestration | 573.8 |
|  | Acute intrahepatic cholestasis | 576.8 |
| Renal | Hematuria | 599.7x, 791.2 |
|  | Proteinuria | 791.0 |
|  | Nephrolithiasis | 592.x, 594.x, 788.0, 274.11 |
|  | Polyuria | 788.42 |
|  | Renal insufficiency | 593.9, 585.9 |
|  | Glomerulonephritis | 580.x, 582.x |
| Pulmonary | Pneumococcal pneumonia | 481 |
|  | Pulmonary embolism | 673.2x, 673.8x, 415.1 |
|  | Pulmonary hypertension | 416.0 |
|  | Acute Chest Syndrome | 517.3 |
|  | Asthma | 493.0–493.9 |
| Spleen | Splenic sequestration | 289.52 |
|  | Hypersplenism | 289.4 |
| Other | Retinopathy | 362.1 |
|  | Leg ulcers | 707.1x |
|  | Obstructive sleep apnea | 327.23 |
|  | Aseptic (Avascular) bone necrosis | 733.4 |
|  | Deep Vein Thrombosis/Thrombosis | 444.x, 451-453, 671.3, 671.4, 671.9 |
|  | Hand foot syndrome (Dactylitis) | 282.62, 282.64, 282.69 |
|  | Priapism | 607.3 |
|  | Osteomyelitis | 730.0, 730.1, 730.2 |
|  | Fever | 780.60 |
|  | Infectious and parasitic diseases | 001-139 |
|  | Aplastic crisis | 284.9 |
|  | Hyperhemolysis  (delayed hemolytic transfusion reaction) | 999.63, 999.73, 999.78, 999.85 |
